# Navigating the Prescription Drug Information System

**DOI:** 10.1002/cpt.70401

**Published:** 2026-07-25

**Authors:** Irina V. Wang, Courtney Davis, Robin Forrest, Avi Cherla, Maximilian Siebert, Janice Cowden, Sophia Walker, Tamar Krishnamurti, Florence T. Bourgeois, Steven Woloshin, Anita K. Wagner, Huseyin Naci

**Affiliations:** ^1^ Pharmaceutical Policy Lab, LSE Health The London School of Economics and Political Science London UK; ^2^ Department of Global Health and Social Medicine King's College London London UK; ^3^ Lisa Schwartz Foundation for Truth in Medicine Norwich Vermont USA; ^4^ Department of Health Policy The London School of Economics and Political Science London UK; ^5^ Division of Health Policy and Insurance Research, Department of Population Medicine Harvard Medical School and Harvard Pilgrim Health Care Institute Boston Massachusetts USA; ^6^ Harvard‐MIT Center for Regulatory Science Harvard Medical School Boston Massachusetts USA; ^7^ Patient Advocate North Port‐Sarasota Florida USA; ^8^ Patient Partner Cambridge Massachusetts USA; ^9^ Division of General Internal Medicine University of Pittsburgh Pittsburgh Pennsylvania USA; ^10^ Computational Health Informatics Program Boston Children's Hospital Boston Massachusetts USA; ^11^ The Dartmouth Institute for Health Policy and Clinical Practice Hanover New Hampshire USA

## Abstract

Information on new prescription drugs is increasingly complex and fragmented, posing challenges for healthcare professionals, patients, and payers. Clinicians require concise, actionable guidance to support prescribing decisions, while patients seek to balance benefits and harms when making decisions aligned with their treatment goals. Payers need comparative effectiveness data to inform coverage decisions that shape access and resource allocation. However, inadequacies in drug information communication often lead to overestimation of benefits and underestimation of harms. In this White Paper, we explore how information about newly approved drugs is generated, disseminated, and interpreted. We examine the information needs of healthcare professionals, patients, and payers and the influence of regulatory standards on available evidence. Focusing on regulated communication channels, such as prescription drug labeling, medication guides, and clinical trial snapshots, we highlight the foundational role of regulatory agencies in setting evidence standards and legitimizing drug information. We also consider alternative information sources, including pharmaceutical advertising, which coexist with regulated content. Against a backdrop of increasing uncertainty of drug approval evidence, we identify documented principles that regulators can adopt to meet drug information needs. Recommendations for improvements aim to better serve patients, support clinical decision making, and promote ethical healthcare resource allocation. While centered on the US context, the paper draws parallels with the European information landscape.

Information on prescription drugs has become increasingly complex and fragmented, making it challenging to navigate for everyone who needs drug information. Healthcare professionals seek practical, succinct guidance for prescribing decisions that best meet the needs of their patients.^1^ Patients desire understandable, jargon‐free information to help them make healthcare choices that are in line with their treatment goals and preferences,^2^, ^3^ yet are often confronted with an array of information sources, from lengthy, technical clinical data summaries to emotionally charged or inaccurate advertisements.^4^ Meanwhile, payers require reliable data on comparative effectiveness to inform coverage decisions that ultimately determine both individual access and the allocation of healthcare resources across populations.^5^

Patients frequently overestimate benefits and underestimate harms of medicines,^6^ likely due to inadequacies and inconsistencies in communication. When clinical trial data are filtered through multiple layers of interpretation—from regulatory information to pharmaceutical marketing and social media influencers—the original safety and efficacy evidence can become distorted in ways that systematically favor optimistic assessments while obscuring important uncertainties and limitations.^7^

In this White Paper, we discuss how information about newly approved prescription drugs is created, disseminated, interpreted, and used by key stakeholders. First, we consider the distinct information needs of healthcare professionals, patients, and payers in context. We then explore where information on new drugs originates, describing how commercial incentives and evolving regulatory standards shape what evidence is available. The paper focuses on regulated communication channels given regulatory agencies' foundational role in setting evidence standards for marketing authorization, their unique position in scrutinizing and legitimizing the information available on new medicines, and their mission to provide information that is indexed across other channels and serves as the basis for treatment decisions.^8^ Regulated communication tools are assessed, including prescription drug labeling, medication guides, and clinical trial snapshots.

We also address information sources that exist outside of those issued by regulators, from pharmaceutical company advertising to social media influencers. Finally, we identify evidence‐based principles that regulators can adopt to improve the prescription drug communication system in ways that better serve patients, support clinical practice, and enable equitable and effective allocation of healthcare resources. Although our focus is on the United States, we also draw parallels with the information landscape in Europe.

Throughout, we use various drug examples to illustrate how system‐level issues in information creation, dissemination, and interpretation impede the information needs of healthcare professionals, patients, and payers.

## WHAT ARE THE DRUG INFORMATION NEEDS OF HEALTHCARE PROFESSIONALS, PATIENTS, AND PAYERS IN DECISION MAKING?

When making treatment decisions, healthcare professionals, patients, and payers approach the same drug with different perspectives, timelines, and decision‐making constraints. Yet, all depend on information derived from the same underlying data, especially in the case of newly approved drugs for which there is limited real‐world clinical and patient experience data.

### Healthcare professionals' information needs

Historically, clinicians have been seen as making decisions on behalf of their patients.^9^, ^10^ The professional infrastructure of medicine—from medical education through clinical practice guidelines—developed around the assumption that clinicians would serve as information intermediaries, translating complex scientific evidence into accessible treatment recommendations.^11^ Thus, today's provision of regulated information was designed around healthcare professionals as the intended audience. While healthcare professionals continue to play a pivotal role for patient guidance in the clinical setting, the increasingly complex information environment is evolving toward shared decision making—though ultimate legal responsibility for the prescribing decision remains with the physician.^12^, ^13^

To make informed decisions with patients, physicians themselves must navigate a complex landscape of information. This is especially difficult in therapeutic areas that are undergoing rapid change, with new treatments receiving regulatory approval—as has occurred in Alzheimer's disease, where the FDA has approved three new drugs since 2020 (one of which was subsequently withdrawn from the market by its manufacturer). In such a context, the information demands placed on prescribing physicians in order to fulfill clinical responsibilities and contribute effectively to shared decision making with patients and their caregivers are substantial.^14^ They must have access to information on therapeutic benefits presented alongside harms, comparative effectiveness, patient selection criteria, burden of treatment, regular monitoring needs, and acknowledgment of what remains unknown. To help patients weigh benefits against harms in the case of Alzheimer's disease, physicians must be aware of the long‐standing controversies that have surrounded new drug approvals—namely, the likely limited ability of current drug therapies to prevent cognitive decline in the face of serious risks of brain swelling and bleeds.^15^, ^16^, ^17^

Physician information needs are compounded by the complexity of the clinical setting. Time pressures in clinical encounters demand that essential information be readily accessible and efficiently communicated to providers in a format that supports facilitation of patient understanding and engagement in a shared decision‐making context.^18^

### Patients' information needs

When making treatment decisions, questions for patients and their family members and caregivers are more personal and concrete than those facing the physician.^19^, ^20^, ^21^ For example, for a patient starting to take antidepressants for the first time, important questions include: Would this treatment meaningfully reduce depressive symptoms? What is the likelihood of experiencing side effects? What are the implications of weight gain? How would the balance of potential benefits and harms impact quality of life, and which aspects of life? Patients need information about clinical significance, including whether observed benefits translate into meaningful improvements in their daily lives, when, and how long.^22^ Patients and physicians may weigh the balance of benefits and harms differently, and patients themselves vary considerably in how they assess the tradeoffs between the risks, the potential short‐term and longer‐term gains of treatment, and the burdens of side effects. These needs map onto the same categories required for healthcare professionals.

Prescription medication decisions can be stressful for patients, especially when the right choice is not obvious. Heightened emotion can affect deliberative processing of available information, while insufficient information or poor patient‐provider communication can lead to feelings of mistrust. Patients often process health information over time, in consultation with others, such as family and caregivers. This challenge is compounded in therapeutic areas where multiple treatments exist, as is the case in depression, where choosing among a broad range of antidepressants requires robust comparative evidence to identify the most appropriate agent for each patient.^23^

Information should be accessible across diverse educational and sociocultural backgrounds. It is estimated that as many as 77 million US adults would experience difficulties with common health tasks like following a prescription drug label.^24^, ^25^ Multiple presentation formats—visual, narrative, and quantitative—can accommodate different learning preferences and language capabilities. Although they are important to consider, literacy levels are unreliable surrogates for comprehension, as materials can appear simple yet fail to convey critical information or support meaningful decision making. As a result, messages need to be tested and tailored in order to make sure they work for an intended audience.^26^

### Payers' information needs

Meanwhile, insurance companies face their own set of questions. Is a drug recently approved by the FDA worth the additional cost compared to existing treatments given its clinical benefits? Should it be covered for all patients for whom the drug is approved, or only those meeting specific criteria? What are clinically and ethically defensible criteria for limiting drug coverage to those who could benefit from the drug? How would coverage affect spending, especially when drugs are very expensive and/or used for indications that affect large numbers of people?^27^ Payers function as gatekeepers for patient access to prescription drugs through formulary decisions that determine coverage, cost‐sharing requirements, and utilization management policies.^28^ They make evidence‐based assessments about the value of new treatments relative to existing alternatives and resources in the healthcare system. Unlike clinicians who consider primarily individual patient benefit, or patients who focus on personal outcomes, payers must consider both individual member and population‐level impacts and trade‐offs in allocating resources across services and patient populations.

Accordingly, payers need information to make decisions about whether to cover a new prescription drug in their formularies, among therapeutic choices, which prescription drugs they cover, how much to pay for them, and how to structure coverage policies that balance appropriate access with system‐wide sustainability.

In some high‐income countries outside the United States, health technology assessment (HTA) bodies have a statutory responsibility to advise payers and to some extent address these information needs.^5^ In the United States, the Institute for Clinical and Economic Review (ICER)—an independent, non‐governmental organization—partially takes on this role by conducting value assessments of selected new treatments.^29^ However, its recommendations are not binding, and payers are not required to adopt them. Instead, pharmacy and therapeutics committees of public and private payers in the United States are responsible for these assessments. Their review processes often lack transparency and may proceed with incomplete evidence about clinical and long‐term outcomes.

The connection between payer decisions and patient access also deepens the ethical dimensions surrounding information needs. There can be significant public and political pressure surrounding reimbursement for FDA‐approved treatments, wherein patients and advocacy groups perceive coverage denials as arbitrary insurance company policies made in the interest of saving costs. The complexity of these decisions increases even further as new treatments often come with premium pricing that may or may not reflect proportional improvements in patient outcomes.^30^

Healthcare professionals, patients, and payers each bring distinct perspectives, constraints, and decision‐making contexts that require unique approaches to information presentation and communication. Understanding this web of interconnected needs raises critical questions: What information is available on newly approved medicines, when does it become available and in what form? How do the commercial, regulatory, and professional forces that shape information creation affect the timing, quality, completeness, and accessibility of information about the evidence available to support prescription drug decisions?

## INFORMATION CREATION: WHAT INFORMATION IS AVAILABLE ON NEW DRUGS AT THE TIME OF APPROVAL?

Before patients, physicians, and insurance companies learn about new drugs, teams of researchers spend years conducting clinical trials and collecting other data that determine the evidence on new drugs' benefits and harms. The system in which the information comes to exist is complex. Understanding the origins of information on newly approved drugs requires tracing a web of actors, incentives, and evolving regulatory standards that fundamentally shape what is known—and what is not known—about new drugs.

### Regulatory evidence standards for drug approval

Regulatory authorities serve as an important gatekeeper to the pharmaceutical market.^31^ Drug companies cannot market their products without receiving regulatory approval, and the evidence standards set by regulatory agencies fundamentally shape the quantity and quality of available evidence on new drugs.^32^

Regulators require manufacturers to show efficacy and safety of a drug before marketing.^33^ Under the requirement, a drug's clinical efficacy must be demonstrated in an adequate, well‐controlled trial^34^ showing a statistically significant difference in treatment outcomes from patients treated with the drug compared to control. In addition to evaluating the balance of benefits and risks, regulatory assessment considers disease severity and unmet medical need.^35^ Despite never mandating that new drugs have a certain magnitude of clinical efficacy or that they are compared to existing clinical practice alternatives in head‐to‐head trials, regulatory evidence requirements have given rise to the belief that newly approved drugs “work”—that they have been shown to make patients feel better and live longer. That assumption underlies recommending, prescribing, using, and paying for new drugs.^36^

Starting in the 1980s, the FDA has been using special programs to expedite the US drug development and review processes for drugs to treat serious conditions with an unmet medical need (similar programs exist in Europe). While regulatory flexibilities have benefits when done well, they have also led to the approval of medicines with significant gaps in evidence of clinical benefit and harm.^37^, ^38^ Clinical studies supporting regulatory approval of new drugs increasingly involve small, nonrepresentative groups of patients, and usually omit comparisons to established standards of care.^39^, ^40^ Compared to 1995–1997, the proportion of approvals supported by at least two pivotal trials decreased from over 80% to about 50% by 2015–2017, and the proportion of approvals supported by only single‐arm trials increased from about 4% to 17% in 2015–2017.^40^ These trends are most evident for cancer drug approvals, which now make up the single largest category of new drug approvals. Between 2000 and 2016, only 51% of trials supporting the approval of cancer drugs were randomized,^41^, ^42^ and single‐arm trials are common in regular approvals of cancer drugs.^43^

Today's regulatory approval increasingly relies on “surrogate endpoints” such as laboratory measurements or imaging results that serve as presumed indicators of expected clinical outcomes. Surrogate endpoints shorten the duration and cost of clinical trials, expediting the development and approval of new medicines. When validated, they can predict clinical benefits of drugs for specific indications. However, most surrogate endpoints have little or no correlation with patient‐centered outcomes, such as how people feel or how long they live.^38^, ^44^, ^45^, ^46^, ^47^ Yet, many new drugs receive approval based on surrogate endpoints, contributing to a system where regulatory approval signals effectiveness that may not exist in any meaningful clinical sense. For example, among new cancer drugs approved by the FDA between 2006 and 2023, most were approved on the basis of surrogate endpoints and fewer than a third had evidence of overall survival benefit.^48^ Notably, this pattern is not explained by accelerated approvals, which explicitly allow market entry based on surrogate endpoints “reasonably likely” to predict clinical benefit, since these accounted for only 29% of cancer drug approvals in that period.^48^ Beyond cancer, surrogate endpoints used to support FDA approval of drugs for nononcologic chronic diseases similarly lack high‐strength evidence of associations with clinical outcomes.^49^

The increase in the use of expedited regulatory approvals has led to a shift in evidence generation from the pre‐approval to post‐approval phase. Regulators stipulate that evidence of clinically meaningful benefits be shown *after* marketing for drugs that are approved through expedited programs. However, this often does not happen, as postapproval studies are frequently delayed.^50^ Even when these studies are completed, they tend to rely on the same surrogate endpoints used in preapproval trials and fail to address the information needs of patients and other stakeholders. In some cases, postapproval trials show that the drug in its approved indication is not effective, which occurred for approximately one‐quarter of cancer drug indications granted accelerated approval by the FDA between 2012 and 2020.^51^ When evidence indicates that drugs lack clinical benefit, they may remain on the market for extended periods.^52^ Withdrawal can be slow for several reasons: regulators must follow due process, evidence may be ambiguous, patients and physicians may believe the drug is working as intended, and companies often dispute the interpretation of trial results.^53^ In response, there have been growing efforts in recent years to expedite the withdrawal of drug indications with failed postmarketing trials.^54^

There are important health and economic consequences of the early availability of drugs that later prove ineffective. In 2008, the FDA granted accelerated approval to bevacizumab (Avastin) for the treatment of metastatic breast cancer based on initial studies showing only a modest effect on tumor growth—a surrogate endpoint. The confirmatory trial did not show that bevacizumab extended the lives of women with metastatic breast cancer, and the women taking it risked life‐threatening side effects. The FDA ultimately revoked its approval in 2011.^55^ Similarly, hydroxyprogesterone caproate (Makena) received accelerated approval in 2011 to reduce the risk of preterm birth in women with a prior preterm delivery, based on its ability to raise progesterone levels—again, a surrogate endpoint. The confirmatory trial found no meaningful benefit in neonatal outcomes or reduction of preterm birth, and the FDA withdrew approval in 2023.^56^ In both cases, patients were exposed to the risks associated with treatments that lacked evidence of clinical benefit, sometimes for years, due to the system permitting extended market presence in the absence of confirmatory evidence.

Postmarketing generation of evidence extends to safety. Regulators have pharmacovigilance systems to incentivize the generation of information on drug safety after market entry, enabling continuous monitoring of the risks of medicines.^57^ Such research is essential to identify safety signals when drugs are used in real‐world settings in patient populations that may not represent those included in initial clinical studies supporting regulatory approval. Evidence generated in postmarketing safety studies may prompt regulators to issue safety advisories, which are notifications to prescribers or the public about a potential or confirmed drug risk.^58^ For example, drugs approved through expedited programs have been shown to have more safety events during the postmarketing period compared to nonexpedited drugs.^59^

## INFORMATION DISSEMINATION: HOW ARE REGULATORS CURRENTLY COMMUNICATING ABOUT PRESCRIPTION DRUGS?

Regulators determine the evidence required to authorize the marketing of a new drug. With respect to communication, the FDA is “responsible for advancing the public health… by helping the public get the accurate, science‐based information.”^60^ This mandate poses the challenge of communicating continuously with a varied audience, including “the general public (patients, consumers, and lay care givers), medical professionals (individuals, institutions, and associations), manufacturers, producers, distributors, retailers, advocacy groups, government (state, local, national and international), and other non‐government organizations.”^61^ Thus, the same source information (clinical trial data on benefits and harms, associated uncertainties, and any other available evidence, including new evidence that emerges over time) should be routed through multiple communication channels, each tailored to different audiences and actors.

In practice, several regulated information formats exist: (1) prescribing information (labeling) aimed at healthcare professionals, (2) medication guides and information aimed at patients, and (3) drug trial snapshots (simplified summaries) available to both.^59^ Similar regulated information sources exist in Europe, like the Summary of Product Characteristics (SmPCs) aimed at healthcare professionals, and medicines overviews available on the European Medicines Agency website for the general public.

In this section, we examine the intended aims of existing regulated information formats. While these formats largely achieve what they were designed or legislated to do, the evidence suggests that this is not enough to meet the information needs of prescribers, patients, and payers.

### Regulated information for healthcare professionals

FDA‐approved product labeling is the primary mechanism for the FDA to communicate with prescribers. The objective of FDA‐approved product labeling is to ensure that healthcare professionals have access to accurate, science‐based information that supports the safe and effective use of a drug. FDA‐approved labeling is lengthy and difficult to navigate. The document follows a standardized 17‐section format, with clinical efficacy results summarized in section 14. Notably, safety data are summarized in a separate section. This structure is mandated across all prescription drugs, ensuring some predictability for clinicians who have learned to navigate these documents as part of their professional training.^62^, ^63^ The Prescribers' Digital Reference^64^—a compendium of drug labels—reaches thousands of clinicians, and other digital sources like UpToDate^65^ include labels in their information syntheses. As labels are a primary source of information, any limitations or omissions in FDA‐approved labeling cascade through the healthcare system, influencing clinical decision making at multiple levels and providing a basis for drug claims in advertisements.

In Europe, the Summary of Product Characteristics (SmPC) forms the basis of information for healthcare professionals on how to use a medicine safely and effectively. Structured similarly to the FDA label, it shares many of the same limitations; an analysis of European regulated information sources for anticancer drugs found that scientific concerns about the reliability of evidence were rarely communicated to clinicians,^66^ mirroring findings that show FDA labels for new cancer drugs reported fewer than half of the uncertainties that FDA reviewers themselves identified as important to the approval decision.^67^

In both the United States and Europe, labeling is initially written by pharmaceutical companies based on templates and standards developed by regulators and subsequently assessed, reviewed, and ultimately approved by the regulator. The approved labeling is intended to reflect the available evidence on the medicine. Labels provide extensive detail about dosing regimens, contraindications, and adverse reactions—all information essential for safe prescribing. However, the clinical studies sections have important limitations in the provision of quantitative benefit data that would help healthcare professionals judge how well the drug actually works for patients.^68^ An earlier systematic evaluation of cancer drug labels found inconsistent reporting of overall survival data: in some cases, survival outcomes were presented only in tables or figures without accompanying qualitative interpretation, whereas in others, labels included narrative statements referencing the statistical significance of observed survival benefits.^66^

Labeling often reports clinical trial results based on surrogate endpoints, as these often form the basis of regulatory approval decisions, without offering guidance on how these measures should be interpreted and communicated. In oncology—the main therapeutic area of new drug approvals—endpoints such as progression‐free survival (PFS) or objective response rate (ORR) are commonly used. PFS is generally misinterpreted as a proxy for overall survival,^69^, ^70^ despite evidence that improvements in PFS do not necessarily translate into longer life.^71^ Similarly, ORR, which reflects tumor shrinkage, may imply benefits in survival or quality of life that are not substantiated by trial data.^72^ Crucially, drug labels do not indicate whether the surrogate endpoints used in pivotal trials have been evaluated for their association with overall survival or other clinically meaningful outcomes,^73^ leaving prescribers without essential information for evidence‐based decision‐making.

Despite growing recognition of the importance of data on patients' experiences, perspectives, and preferences, particularly in the form of patient‐reported outcomes, they remain infrequently collected in clinical trials supporting new drug approvals.^74^ In oncology, even when patient‐reported outcomes are evaluated, they are rarely incorporated into labeling, likely reflecting limitations in the quality, consistency, and relevance of the data collected.^75^ Organizations like the International Consortium for Health Outcomes Measurement (ICHOM) are developing standards for measuring patient‐reported outcomes.^76^ There are also recent regulatory efforts to support the generation of patient experience data.^77^, ^78^

A key concern is the limited communication of uncertainties about a drug identified by drug regulators during the approval process. Uncertainties exist with respect to generalizability of evidence, long‐term outcomes, benefit–risk balance, and clinical benefit and its magnitude. These uncertainties are often not reflected in the labeling intended for prescribers.^66^, ^79^ A lack of information creates a false sense of certainty about drugs with benefits and harms that may be far more uncertain than prescribers realize. Indeed, surveys suggest that physicians often mistakenly assume FDA approval is based on robust evidence, thereby overestimating the rigor of the data supporting drug approvals.^80^, ^81^

Despite these substantial insufficiencies, the FDA arguably already has the means to improve its labeling information. FDA review documents, available through the Drugs@FDA database, contain detailed clinical trial data along with information about how reviewers made their decisions about whether a drug provides meaningful benefit. FDA reviewers with extensive expertise assess the evidence to produce comprehensive evaluations that address precisely the questions that patients and clinicians need answered.^82^ These documents contain clinical trial results, detailed statistical analyses, and reviewers' assessments of uncertainty. In fact, the FDA has recently adopted a structured benefit–risk assessment framework, which explicitly considers these uncertainties in the evidence base and summarizes them in a tabular format. However, most of the information in these internal assessments does not appear on labels.^67^, ^83^ Because they were not intended for communication with clinicians and patients, a typical FDA review document can be hundreds of pages long, with technical language and complex formatting.^82^

For patients, the implications are profound. When clinicians lack access to quantitative benefit data or awareness of important uncertainties, their ability to engage in meaningful shared decision making is compromised. Patients may receive recommendations from their clinicians who are overly confident based on incomplete regulated information, undermining patients' autonomy and the informed consent that effective decision making requires. In cases where drug indications are withdrawn after post‐approval studies show harm, clinicians need to stop prescribing what they had previously recommended with confidence,^84^ which can have lasting effects on patient‐physician trust.

### Regulated information for patients

Patients need information on both what is known and the limits of current evidence on their medications. When patients collect their prescriptions at the pharmacy counter, they encounter regulated information in the form of Medication Guides. Medication Guides are standardized documents that are intended to convey serious risks associated with certain prescribed medicines. These documents, which will accompany all prescription drugs in the future,^85^ are the FDA's direct line of communication with patients. Given their primary focus on the safe use of medicines, the content of Medication Guides reveals an asymmetry in how regulated information treats benefits vs. risks. Similarly, in Europe, the patient information leaflets that accompany each medicine currently lack information on drug benefits and uncertainties.

The Medication Guide for antipsychotic aripiprazole (Abilify) exemplifies this pattern. It focuses exclusively on potential harms, providing detailed lists of side effects without the likelihood or magnitude of benefits that might justify accepting these risks. A patient reading about “nausea, vomiting, constipation, headache, dizziness, anxiety, [and] insomnia” has no way to understand that some of these effects occur in most patients while others affect fewer than one in a hundred. This asymmetrical emphasis on risk assumes that patients need protection from harm but does not empower them with the complete information required to autonomously balance risk against known and unknown clinically significant benefit.

The frequent omission of benefit information from patient‐facing materials—which is by design—creates a paradox: the people who must live with the consequences of treatment decisions receive the least information about evidence on the magnitude and nature of treatment benefits. Patients may understand that a medication can cause nausea or dizziness but have no sense of how much improvement to expect, how likely they are to experience benefits, or how long benefits might take to emerge and last. The absence of information on benefits (and the uncertainty surrounding it) may lead patients to overestimate the effectiveness of new treatments. Perhaps most deeply concerning is when a drug with uncertain benefits is given to a patient whose remaining life is measured in weeks.^86^, ^87^, ^88^ In oncology, such use of new drugs has been termed “desperation oncology”^89^ as it wastes individual resources, societal resources, and the precious remaining time that patients may otherwise choose to spend outside of clinical settings.^90^

For healthcare professionals seeking shared decision making, the systemic information asymmetry hinders comprehensive communication in individual healthcare encounters when clinical trial source data and FDA assessments of source data should result in regulated information that facilitates informed decision making.

### Drug trial snapshots

Launched in 2015, Drug Trial Snapshots were developed in response to a legislative requirement to make information about clinical trial diversity easily accessible. They include visual summaries of demographic characteristics and key efficacy data from pivotal clinical studies supporting drug approvals. Infographics highlight the age, sex, race, and ethnicity of trial participants alongside basic information about how the studies were conducted and what they found.^91^

Despite their significance highlighting critical gaps in clinical trial diversity, the snapshots are not produced for all drug approvals. They capture data only from the initial approval studies, not subsequent trials that may support use for additional indications or populations. As for their application in medical decision making, the FDA explicitly states: “Do not rely on Snapshots alone to make decisions regarding medical care,” as they do not communicate benefit–risk assessment information or provide guidance on weighing trade‐offs between therapeutic effects and potential harms.^91^

Similar to Medication Guides, Drug Trial Snapshots present clinical trial results using language that is prone to misinterpretation. Although communicating key uncertainties falls outside their stated purpose, the absence of information about study design, choice of endpoints, and other methodological features leaves users without context that is important for interpreting the results. The snapshot for tarlatamab‐dlle (Imdelltra),^92^ approved for small cell lung cancer in 2023, exemplifies these limitations. The pivotal trial did not include a control group, yet this is not disclosed. While the snapshot mentions that the product received accelerated approval, it does not do so in a way that acknowledges the heightened uncertainty about clinical benefit.^93^

In the European Union, EMA's Medicine Overviews serve a similar function by providing a publicly accessible summary of medicines authorized in the European Union. Medicines Overviews include approved indications, mechanism of action, key clinical study findings, and evidence on effectiveness, safety, and potential side effects.^94^ However, some of the same concerns are noted as with the Drug Trial Snapshots, including potentially misleading language describing surrogate endpoints, and no acknowledgement of an absence of evidence with respect to endpoints that matter to patients (e.g., whether patients can expect to feel better or live longer).^66^

Regulatory documents like labeling, Medication Guides, and Drug Trial Snapshots are a crucial part of the drug information communication system. They are designed to satisfy regulatory and legislative mandates that do not adequately reflect the actual user information needs. The FDA already possesses the underlying information in its review documents, which contain the clinical trial detail, the uncertainty assessments, and the benefit–risk analyses that are largely absent from regulated communications. Rather than treat their communication channels and touchpoints as compliance obligations, regulators have the opportunity and authority—and, we would argue, the responsibility—to equip prescribers, patients, and payers with the information that each of them requires to make meaningful decisions.

### Regulated information and payers

Although the FDA maintains no formal information pathway specifically designed for payers, regulated information forms the foundation for most coverage and reimbursement decisions. Payers rely on FDA‐vetted clinical trial data when making formulary decisions, and they require different information than what regulatory approval provides. While the FDA evaluates whether a drug demonstrates sufficient efficacy and acceptable safety for marketing approval, payers' concerns include comparative effectiveness, cost‐effectiveness, and population‐level budget impact (e.g. Does this drug work better than existing alternatives? Is the improvement worth the additional cost?) When the FDA approves a drug based on statistical significance or marginal clinical significance that reviewers questioned internally, this uncertainty rarely reaches payer decision makers in accessible form.

Health technology assessment (HTA) bodies produce complementary evidence on new drugs to inform value assessments and meet payer information needs. These evaluations increasingly occur soon after regulatory approval and rely largely on the same evidence base as regulators', meaning that regulatory standards influence HTA practices. Given HTA's focus on comparative value, a diverse range of methods is employed, including network meta‐analyses and economic evaluations, which often require strong assumptions due to limitations in the underlying evidence.^95^ Patients and healthcare professionals may perceive coverage denials as arbitrary insurance company decisions, unaware that regulatory approval and coverage determinations address fundamentally different questions. When the FDA granted controversial accelerated approval in 2021 to aducanumab (Aduhelm), some insurers refused coverage, arguing that the evidence underlying approval did not demonstrate meaningful clinical benefit and that they considered the drug “experimental.”^96^ The standoff left patients caught between an FDA‐approved treatment and insurance companies unwilling to pay for it—a situation that persisted until the drug's manufacturer, Biogen, withdrew the drug from the market in 2024.^97^ Similar dynamics have emerged around accelerated approvals for rare disease treatments like spinal muscular atrophy and Duchenne muscular dystrophy therapies, where payers attempt to restrict reimbursement based on limited clinical efficacy evidence, only to face pressure from patient advocacy organizations.^98^

## INFORMATION SEEKING AND INTERPRETATION: WHERE DO PEOPLE TURN FOR INFORMATION BEYOND REGULATED SOURCES?

Most patients may not understand information in the Medication Guide—or they may not find it useful.^99^, ^100^ Meanwhile, they encounter social media influencers sharing emotional stories about treatment narratives, journalists framing new drugs as revolutionary, and experts debating whether clinical benefits justify the risks and costs. These alternative sources, while often more accessible and emotionally resonant than regulated materials, introduce layers of complexity, bias, and potential misinformation into an already fragmented information system.

Prescribers face parallel challenges, turning to journal articles, medical conferences, clinical practice guidelines, and pharmaceutical industry representatives to learn about new drugs. Both patients and physicians find themselves navigating a complex web of sources that interpret, filter, and sometimes distort the original clinical study information that underpinned the regulatory decisions.

### The professional information interpretation system

When learning about new drugs, healthcare professionals often turn to the medical literature. Despite major progress in clinical study registration and reporting in recent years, clinical studies with positive results still appear faster in the literature than studies with negative findings.^101^, ^102^, ^103^ When published, articles often have biased or incomplete reporting of benefits and harms.^104^ Even systematic reviews of clinical studies are not always informative and their methods are not fully reproducible.^105^

Another important source of information is clinical practice guidelines, which represent the most systematic attempt by the medical profession to interpret available data on existing treatment options, including regulatory evidence, for clinical practice. Professional organizations like the National Comprehensive Cancer Network develop treatment recommendations based on published clinical trials, regulatory submissions, and expert consensus. However, the quantity and quality of evidence underpinning clinical practice guideline recommendations is variable.^106^, ^107^, ^108^, ^109^

Academic conferences are also a crucial venue for information interpretation, where clinical trial results are presented, debated, and contextualized. Clinical studies presented in conferences are not always published in peer‐reviewed journals or are published with delay depending on their outcomes (with studies reporting positive results appearing faster than those reporting less favorable results).^110^

Unfortunately, none of these processes are immune to the conflicts of interest that affect information creation and dissemination.^111^ Industry funding of clinical studies,^112^ of many professional organizations,^113^ and of guideline development committees^114^ means that even professional interpretation may reflect commercial interests rather than scientific evidence. Even when peer‐reviewed articles reporting clinical trials may be biased in favor of industry.^115^

In a long‐standing practice, pharmaceutical company representatives also engage directly with clinicians to increase the prescribing and sales of their drug.^116^ In one‐to‐one detailing visits, representatives provide physicians with product information and build sustained relationships over time,^117^, ^118^ and the provision of free drug samples often familiarizes prescribers with new, most‐expensive products.^119^ A study of consecutive sales visits across the United States, Canada, and France found that serious adverse events were rarely mentioned, even where regulation requires “fair balance” between benefit and harm information.^120^, ^121^ These interactions between industry and prescribers extend to sponsored events and continuing medical education programs.^122^ The information clinicians receive through these channels is shaped by the companies' goal to increase sales, which compounds the optimistic biases already embedded in the original clinical trial data.^123^

It is crucial that regulated drug information—ideally based on sound clinical trial evidence—remains the primary reference source, and that it is widely disseminated and consistently referenced wherever possible.

### The direct‐to‐consumer interpretation industry

Meanwhile, patients and the public encounter a striking amount of consumer‐targeted information. The United States is, besides New Zealand, the only country that explicitly allows direct‐to‐consumer (DTC) prescription drug advertising. Pharmaceutical companies' marketing efforts are substantial as they aim to disseminate information to increase use—and thus sales—of their drugs through news articles, magazine ads, online banners, TV commercials, and social media placements. Pharmaceutical companies spent $6 billion on DTC advertisements of prescription drugs in 2016.^124^ In 2024, the figure was over $10 billion.^125^

Research demonstrates that benefits are rarely quantified in DTC advertisements—and when they are presented, they use exaggerated formats that overstate clinical significance.^4^, ^126^, ^127^, ^128^ Meanwhile, harms are relegated to long lists in small fonts that obscure their actual frequency and clinical importance. Television ads often substitute emotional appeal and lifestyle imagery in the place of condition‐specific factual content—a pattern that has intensified over time despite voluntary industry guidelines.^129^, ^130^ For example, the pharmaceutical company marketing the Alzheimer's drug lecanemab‐irmb (Leqembi) launched an extensive advertising campaign that included a television commercial depicting older adults engaged in meaningful activities with taglines like “You've still got a lot to be, with Leqembi,” accompanied by upbeat music and hopeful imagery.^131^ The ads employ qualitative descriptions of benefits without quantitative data about the modest magnitude of effect, while relegating the serious risk of brain bleeding to fine print. When researchers created mock advertisements that prominently featured quantitative data about severe adverse effects, survey respondents perceived greater risks, found the drug less effective, and were less likely to ask their physician about it or consider using it.^132^ Although the current US administration seeks to limit misleading prescription drug advertising,^133^ results are yet to be seen.^134^

### Information in the digital age

The internet has fundamentally transformed how patients access and interpret health information, creating both democratized access and significant risks for misinformation. Most Americans have for decades been turning to the internet first for health information.^135^ On social media and in online communities, patients share experiences, interpret clinical trial results, and provide emotional support during treatment decisions.^136^, ^137^, ^138^ These platforms and meetups offer something that regulated information sources cannot: peer experience and narrative context that helps patients understand what treatment might actually feel like in daily life.^139^ On the other hand, there is the risk of patients interpreting anecdotal experiences as representative of sound evidence for treatment outcomes.

Algorithmic curation introduces layers of interpretation beyond human control. Search engines and social media algorithms may prioritize content based on engagement rather than accuracy, potentially amplifying emotionally compelling but scientifically questionable interpretations of drug information. Social media influencers on platforms like TikTok have emerged as particularly powerful and problematic interpreters of pharmaceutical information, as exemplified by celebrities promoting drugs like semaglutide (Ozempic) without proper risk disclosures, creating viral trends that can drive off‐label use and shortages of medications needed by patients for their approved indications.^140^ Research shows an increase in the pharmaceutical industry's use of customer engagement strategies, like influencer sponsorship and targeted promotions.^141^

The rapid adoption of large language models (LLMs) and artificial intelligence (AI)‐powered health assistants adds another interpretive layer. While LLMs may offer more accessible explanations of complex medical information, they face significant limitations in pharmaceutical contexts.^142^ Research demonstrates that LLMs consistently overestimate their confidence when verbalizing uncertainty, presenting a critical concern for medical decision making.^143^ The absence of confidence and uncertainty estimations in leading LLMs like GPT‐4 is particularly problematic in healthcare applications where understanding the limits of AI‐generated information is crucial for patient safety.^144^

### News media distortions

News media serve as crucial interpreters of medical research for the general public, yet systematic analysis reveals significant problems in how clinical trial evidence gets translated into news coverage.^145^ A substantial share of news stories fail to quantify benefits and potential harms.^146^, ^147^ Many daily newspaper reporters lack appropriate training in health coverage or statistical interpretation, with surveys showing this as a major challenge in their work. The time pressure to produce compelling stories, combined with a lack of subject‐matter expertise, can create distortions in how clinical trial evidence is interpreted for public consumption—though there is the opportunity to improve reporters' science literacy through low‐cost and simple educational interventions.^148^

Distortions introduced at any point into the web of drug information interpretation can propagate throughout the system. For instance, when creators of information emphasize relative benefits in their marketing materials, journalists may adopt similar framing in news stories.^149^, ^150^ Patients might then arrive at clinical appointments with exaggerated expectations, and physicians may feel pressured to recommend treatments they might otherwise question.

The case of the Alzheimer's disease drug lecanemab shows how distortions introduced in news media can propagate across the information system. The company's original press release announced that lecanemab “reduced clinical decline on the global cognitive and functional scale, CDR‐SB, compared with placebo at 18 months by 27%, which represents a treatment difference in the score change of −0.45 (*P* = 0.00005) in the analysis of Intent‐to‐treat (ITT) population,”^151^ without specifying that the 0.45‐point reduction was on an 18‐point scale. The original finding—a 0.45‐point improvement on an 18‐point cognitive scale of uncertain clinical significance, accompanied by a 12.6% risk of brain swelling and documented cases of fatal brain hemorrhage^152^, ^153^—was filtered through a press release that emphasized relative rather than absolute benefits, and subsequent referential news coverage inherited this framing.

The proliferation of alternative, unregulated interpretation sources highlights the need for system‐wide reform. Patients seeking to make informed decisions about treatments often end up less informed after consulting multiple sources than they would be with access to clear, complete regulatory information that acknowledges uncertainty while providing the quantitative context needed for meaningful risk–benefit assessment.

## RECOMMENDATIONS TO IMPROVE COMMUNICATION OF INFORMATION ABOUT PRESCRIPTION DRUGS

Evidence suggests that there are ways to improve the status quo. To better meet the needs of healthcare professionals, patients, and payers outlined above, we recommend strengthening regulators' role in communication, presenting side‐by‐side data on benefits and harms, communicating uncertainty and gaps in evidence, visualizing comparative data across alternative treatments, testing alternative ways to communicate, and understanding the wider system within which drug information is communicated.

### Strengthen the regulators' role in communication

The broader system requires a fundamental overhaul. Conflicts of interest in the creation and dissemination of drug information are pervasive and well documented.^154^ Yet, current management practices leave substantial room for improvement. The professional information system, comprising academic conferences, peer‐reviewed journal publications, and clinical practice guidelines, requires stronger safeguards for managing conflicts of interest.^155^

The direct marketing practices of industry warrant renewed scrutiny. Industry spending on direct‐to‐consumer marketing, as a proportion of overall marketing budgets, is disproportionately higher for products with limited therapeutic benefit than those with added therapeutic benefit, suggesting that promotional activity is driven by commercial rather than clinical value.^156^ Regulators have statutory authority over misleading advertising, yet enforcement has been limited. Regulatory authority is also limited in coverage: social media posts by influencers who receive payments from the manufacturer of a product they are promoting should be subject to the same regulatory oversight of pharmaceutical advertising as if the company had provided the information directly. Although there is some regulatory precedent for such oversight, it remains limited in scope.^157^

Regulators have an opportunity to correct these distortions in the evidence base, as they have access to more granular clinical trial data than is available to other stakeholders, such as peer reviewers or developers of clinical practice guidelines. However, regulators' engagement in disseminating information has been limited. While much emphasis has been placed on improving transparency by making documentation publicly available,^158^ this is insufficient unless coupled with efforts to make information more visible and understandable to patients, prescribers, payers, and the general public. Accurate information that is difficult to find or understand will not correct distortions that are, by design, easy to encounter and hard to ignore. Changes to regulated information sources such as labeling or medication guides need not extend their length but should prioritize presenting the most pertinent information in a way that facilitates comprehension and decision making.

### Present side‐by‐side data on benefits and harms

Like regulators deciding on drug approvals, prescribers and patients need to weigh the potential clinical benefits of a drug against its potential harms. To do so, they need to see information on clinical benefits next to information on harms.^26^

**Figure 1:**
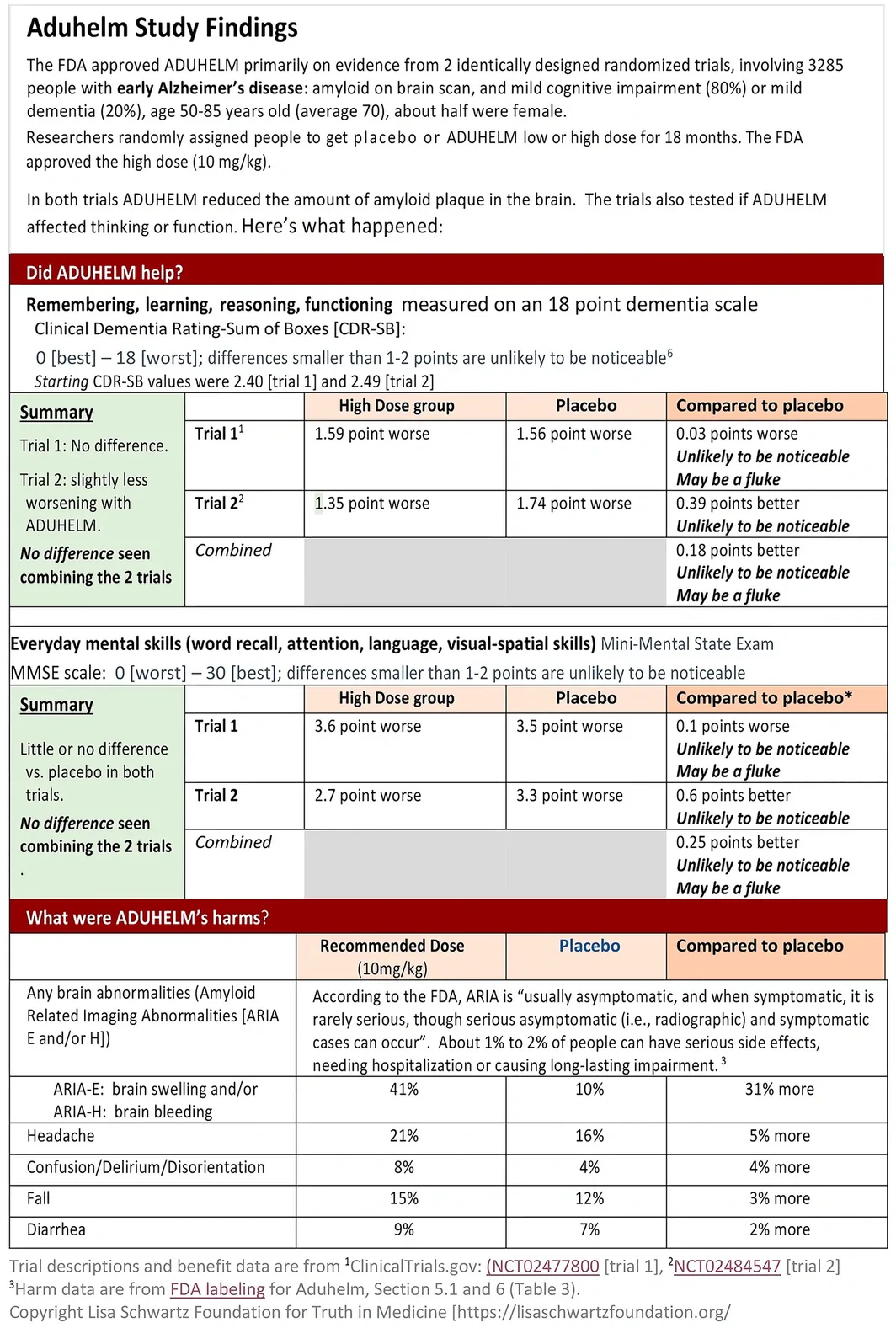
The Drug Facts Box, developed by Schwartz and Woloshin, presents benefit and harm data for a single drug indication in a standardized one‐page format. Randomized trials have demonstrated that consumers who receive the Drug Facts Box showed improved comprehension and made more informed treatment choices than those receiving standard drug information. For aducanumab, the Drug Facts Box (reproduced from Lisa Schwartz Foundation for Truth in Medicine) makes explicit that the two pivotal trials, when analyzed together, showed no meaningful difference from placebo on measures of memory, reasoning, or functioning, while also quantifying serious harms including brain swelling or bleeding.^168^, ^169^ Figure is copyrighted by the Lisa Schwartz Foundation for Truth in Medicine, which is led by co‐author Steven Woloshin, who has given email permission.

Studies of formats such as the Drug Facts Box—a tabular display including numerical benefit and harm data for each indication of a drug—demonstrate that most consumers can understand quantitative information and that it improves decision making (**Figure** **1**).^82^, ^159^

The documented efficacy of well‐executed quantitative tables and graphs reinforces the critical need to provide patients with precise numerical estimates rather than vague qualitative descriptors. When risks are characterized solely through words (e.g., “low probability of side effects”), the information can be interpreted differently by various individuals and fails to provide the specific details necessary for informed decision making.^160^

### Communicate uncertainty and show the gaps in evidence

In keeping with the previous principle, inconsistencies in interpretation can be reduced when numeric expressions of uncertainties (e.g., confidence intervals) accompany verbal probability statements.^160^ Effective uncertainty communication must address not only how statistical uncertainty is expressed but also acknowledge fundamental gaps in available knowledge.^161^ To make informed decisions, clinicians and patients must know what information is fundamentally missing and not yet addressed by high‐quality research. For example, it is important to communicate when a new cancer drug approved on the basis of a surrogate endpoint lacks evidence of improvement in overall survival or quality of life. Studies have found that while communicating statistical uncertainty may slightly reduce perceived trustworthiness of specific numbers,^162^ it may not have a negative impact on the perceived trustworthiness of the information source.^160^ Strategic communication about uncertainties and visible gaps in evidence (e.g., in the form of empty cells in standardized information tables) could even contribute to a sense of transparency that is able to strengthen rather than compromise the credibility of authoritative information sources.

### Show comparative data across multiple alternatives

Consumers of information rarely need to compare a new drug against a single alternative (or no alternative). In most therapeutic areas, new drugs enter crowded markets; a new antidiabetic agent, for example, comes to a therapeutic space with more than 10 drug classes,^163^ while a new antidepressant may enter a field with over 20 established agents.^23^ Even if regulators improve their communication practices and provide clearer information on how a new drug performs relative to the control treatment in its pivotal trial, this will not fully address the information needs in clinical practice where a patient and their clinician need to find the best alternative for an individual patient. Payers must determine whether a new drug offers meaningful benefit over existing alternatives for their insured members and how much to pay for that improvement, given their need for wise financial stewardship of limited resources and population‐level trade‐offs they face.

**Figure 2:**
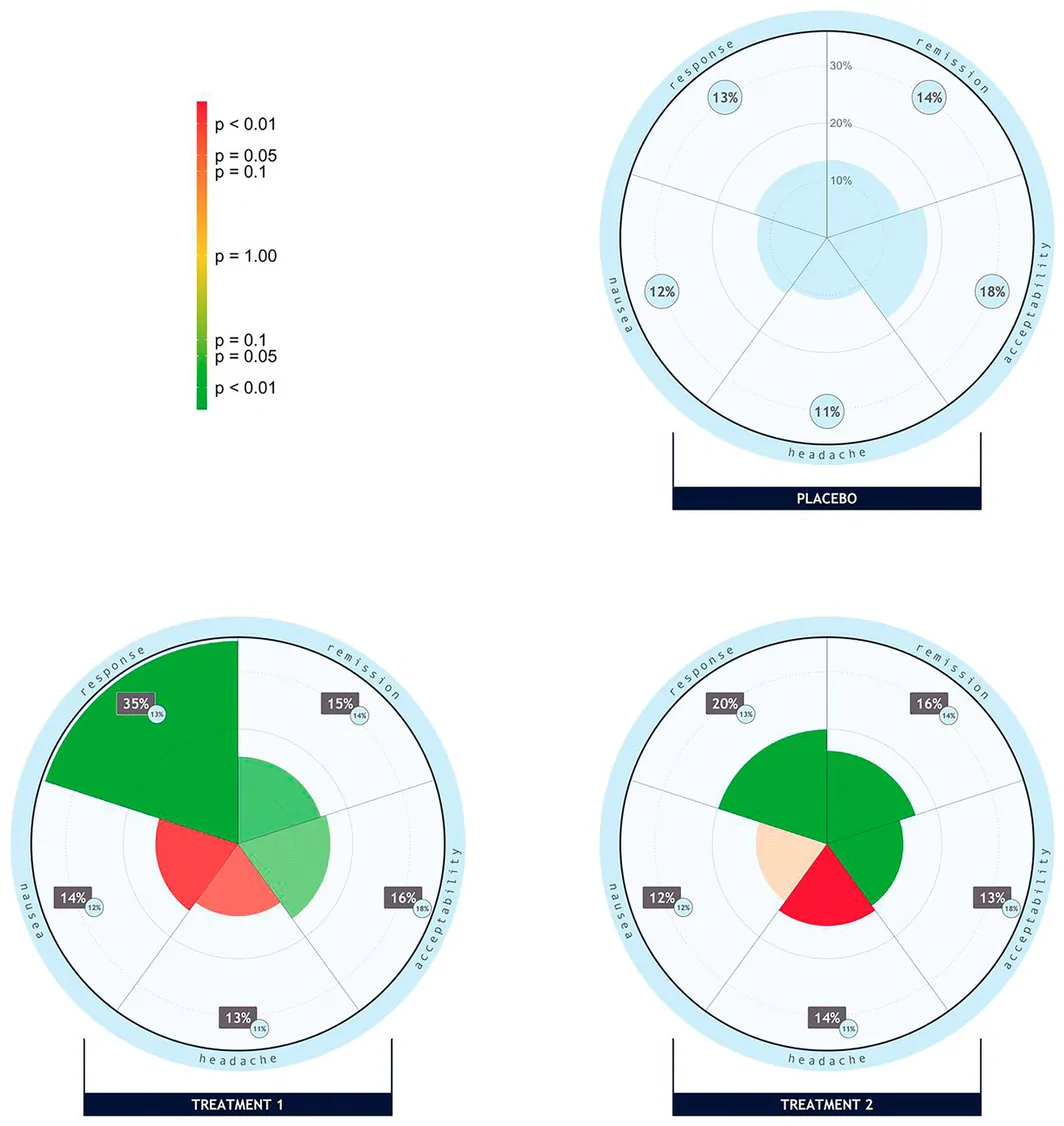
This Vitruvian plot (reproduced from Ostinelli *et al*.^164^) visualizes comparative benefits and harms of different treatments across multiple outcomes.^164^ Color‐coding communicates the strength of statistical evidence or degree of confidence in the evidence, while distance from the center encodes magnitude of effect relative to the comparator for a given outcome. This is a good example of how innovative presentation formats can communicate complex drug information in a more accessible and comprehensive way to a range of decision makers. Figure is licensed under Creative Commons for noncommercial use.

Network meta‐analyses are well suited to address this need, synthesizing the totality of the evidence base to characterize the comparative benefits and harms of multiple treatment options. While these methods are now widely used by health technology assessment bodies, they introduce new communication challenges: a network meta‐analysis comparing multiple treatments across multiple outcomes can generate large numbers of numerical findings that are cognitively demanding to process. Recent efforts to develop and test innovative visual presentations of these complex findings have shown promise in improving comprehension and supporting decision making (**Figure** **2**).^164^, ^165^

These visualization approaches may be particularly relevant for clinical practice guideline developers.

### Design and test alternative ways to communicate information

Effective drug information communication requires empirical testing of messages with intended audiences. The testing process need not be elaborate or expensive; straightforward, practical evaluation methods such as online randomized surveys comparing alternative formats can help determine whether designs successfully convey health messages rather than operating on false assumptions about how people will respond.^26^

### Understand the wider system within which drug information is communicated

Communicating drug information is a “wicked problem” that exists in a complex system of diverse actors.^166^ These actors and dynamics extend beyond the regulators, prescribers, patients, and payers detailed in this paper; the wider system involves policymakers, social media, patient advocacy groups, pharmaceutical companies, and many more. Changing the status quo requires a more comprehensive understanding of interconnected actors, their perspectives, incentives, powers, and constraints.

## CONCLUSION

Creating a drug information system for the 21st century requires more than improving individual messaging—it requires understanding the system and the actors who create, interpret, disseminate, and use pharmaceutical information. In today's rapidly evolving digital landscape, pharmaceutical information is being reshaped by new technologies and communication norms. Patients and clinicians alike now navigate an environment where traditional, regulated information sources co‐exist—and often compete—with a vast array of online content, from social media commentary and influencer marketing to AI‐generated health advice.

Ultimately, there is a mismatch between what clinical studies measure and the information needs of patients, clinicians, and payers. Those needs should be taken into account early on in drug development planning to ensure that evidence generated downstream is complete and relevant. Individuals desire information on comparative effectiveness to guide their healthcare decisions,^1^ yet most trials employ placebo controls rather than active comparators.^39^ While there are ways (e.g., network meta‐analysis) to work around limitations in the evidence base, better trials are needed.^167^

From incomplete trial data to inaccessible information and unregulated sources, there is an ethical question raised about regulators' responsibility in communicating prescription drug information. The FDA's mission explicitly includes “helping the public get the accurate, science‐based information they need to use medical products and foods to maintain and improve their health.”^60^ This obligation extends beyond the binary determination of whether a drug is safe and effective enough for market approval; it demands clear communication about the evidence on the magnitude of benefits, the likelihood of those benefits occurring, the populations in which benefits were demonstrated, and, importantly, what evidence is lacking.

Regulators are uniquely positioned to demand the necessary evidence and improve communication about prescription drugs. Ultimately, the integrity of drug communication depends on a shared information foundation—one that resists commercial, political, and algorithmic bias while empowering patients, clinicians, and payers to make informed decisions. Strengthening the visibility, clarity, and accessibility of regulated, evidence‐based information is not merely a technical challenge but a public health imperative.

## FUNDING

This work was supported by a grant from Arnold Ventures (PI: Florence Bourgeois). AKW received partial grant support from the American Cancer Society for related work.

## CONFLICT OF INTEREST

IVW, CD, RF, AC, MS, JC, SWa, TK, and FB declared no competing interests. SWa reported freelance work as patient editor for the British Medical Journal. AKW reported employment as director of the ethics program at Point32Health. SWo reported serving on the Cochrane Library and JAMA Internal Medicine editorial boards and receiving grant funding from Arnold Ventures. Dr Naci reported receiving grants from the Commonwealth Fund, Health Foundation, UK Research and Innovation, and National Institute for Health and Care Research and personal fees from the World Health Organization and The BMJ, outside the submitted work.
